# The complement system in neurodegenerative diseases

**DOI:** 10.1042/CS20230513

**Published:** 2024-03-20

**Authors:** Jacqui Nimmo, Robert A.J. Byrne, Nikoleta Daskoulidou, Lewis M. Watkins, Sarah M. Carpanini, Wioleta M. Zelek, B. Paul Morgan

**Affiliations:** UK Dementia Research Institute Cardiff, Cardiff University, Cardiff CF24 4HQ, U.K.

**Keywords:** complement, dementia, inflammation

## Abstract

Complement is an important component of innate immune defence against pathogens and crucial for efficient immune complex disposal. These core protective activities are dependent in large part on properly regulated complement-mediated inflammation. Dysregulated complement activation, often driven by persistence of activating triggers, is a cause of pathological inflammation in numerous diseases, including neurological diseases. Increasingly, this has become apparent not only in well-recognized neuroinflammatory diseases like multiple sclerosis but also in neurodegenerative and neuropsychiatric diseases where inflammation was previously either ignored or dismissed as a secondary event. There is now a large and rapidly growing body of evidence implicating complement in neurological diseases that cannot be comprehensively addressed in a brief review. Here, we will focus on neurodegenerative diseases, including not only the ‘classical’ neurodegenerative diseases such as Alzheimer’s disease and Parkinson’s disease, but also two other neurological diseases where neurodegeneration is a neglected feature and complement is implicated, namely, schizophrenia, a neurodevelopmental disorder with many mechanistic features of neurodegeneration, and multiple sclerosis, a demyelinating disorder where neurodegeneration is a major cause of progressive decline. We will discuss the evidence implicating complement as a driver of pathology in these diverse diseases and address briefly the potential and pitfalls of anti-complement drug therapy for neurodegenerative diseases.

## Complement in the brain

**Figure 1:**
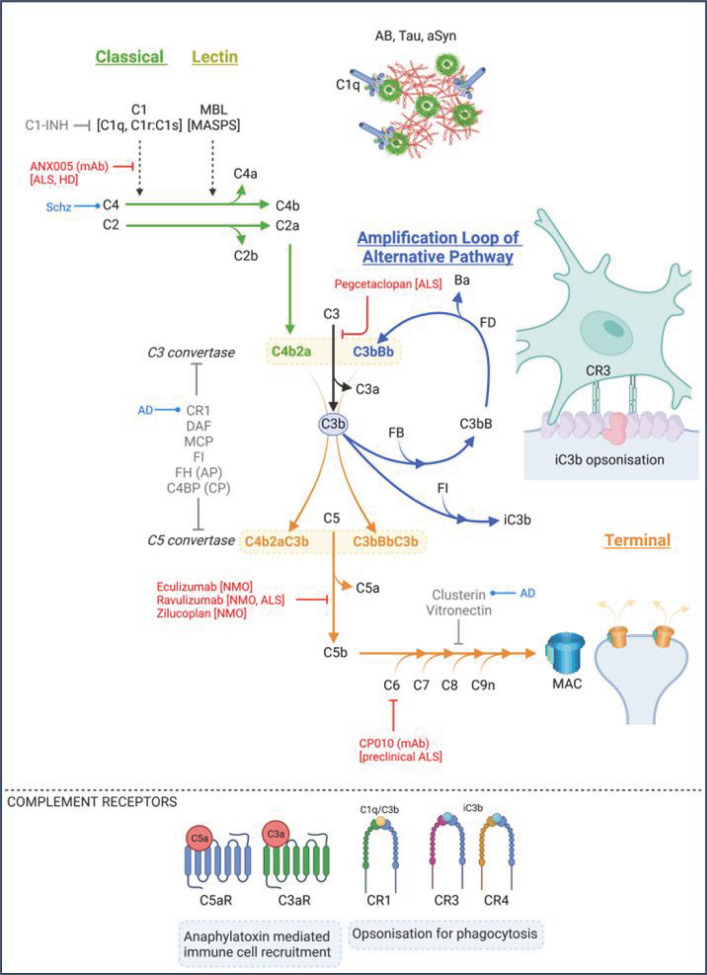
The complement cascade The complement system is activated via the classical, lectin, or alternative pathways. The classical pathway is initiated by C1 binding to antigen-antibody complexes or in brain directly to amyloid, tau or α-Syn aggregates. C1s in the C1 complex cleaves C4 and C2 to form the C3 convertase C4b2a. The lectin pathway is activated by MBL or other lectins binding surface carbohydrates; attached MASPs are activated to cleave C4 and C2 to generate C4b2a. The C3 convertase cleaves C3 to C3b and C3a, the former is further processed by Factor I (FI) to form the opsonin iC3b, a ligand for phagocyte complement receptors CR3 and CR4 to initiate phagocytosis. C3b also feeds back to generate more C3 convertase via the amplification loop; factor B (FB) binds C3b and is then cleaved by factor D (FD) to create the alternative pathway C3 convertase C3bBb. Binding of a further C3b to either C3 convertase creates a C5 convertase which cleaves C5 to initiate the terminal pathway culminating in formation of the membrane attack complex (MAC). The small fragments generated from the C3 and C5 convertases, C3a and C5a, are anaphylatoxins and signal via their receptors, C3aR and C5aR, to recruit immune cells. Numerous cell surface and fluid-phase proteins regulate complement activation and terminal pathway, including C1-inhibitor (C1INH), which inhibits C1s and MASPs, decay accelerating factor (DAF), complement receptor 1 (CR1) and Factor H (FH) which regulate the C3 convertases clusterin, vitronectin and CD59 which control MAC assembly. Complement drugs in clinical/pre-clinical trials are shown in red. GWAS hits in specific diseases are shown in blue. Figure created with BioRender (BioRender.com).

In this brief review we will discuss how complement, essential for immune defence and homeostasis in the healthy brain, can also contribute to pathology in neuroinflammation and neurodegeneration. The basics of the system are summarized in Figure 1. Complement activation in the periphery is a ‘double-edged sword’, functioning to fight infection and resolve inflammation in health but, when dysregulated in disease, causing or exacerbating pathology – and precisely the same applies to complement in the brain. Before considering what complement does in the brain it is first necessary to address some key aspects of brain complement: Are complement proteins present in the brain made locally or acquired from the periphery? How and when is complement activated in brain? How do brain cells respond to complement activation? How is complement regulated in the brain?

Most complement proteins are produced in the liver, secreted into plasma and pass from there into extra-hepatic tissues and organs; local synthesis of complement proteins does occur but is of limited importance in most organs. The brain is different from other organs because it is enclosed in the blood–brain barrier (BBB), restricting the influx of complement proteins and other macromolecules from plasma. The brain, like other organs, must be defended against infection, but the role of complement in this defence was unclear. Early studies demonstrated the presence of complement proteins in cerebrospinal fluid (CSF) and in brain extracts, but the sources of these were uncertain – were they made locally or infiltrated from the periphery [1–3]? Evidence regarding local production of complement in the brain is patchy and often derived from studies in immortalized cell lines of tenuous relevance to primary brain cells. For example, *ex vivo* studies using human brain cell lines or primary cells isolated from postmortem brain demonstrated that almost all complement components, regulators and receptors can be produced by brain cells with neurons, astrocytes, oligodendrocytes and microglia all contributing (reviewed in [4]) (Table 1). Astrocytes are the most abundant non-neuronal cell type in the brain [5], primary astrocytes expressed the regulators CD59, CD55 and CD46, the complement receptors CR1, C3aR and C5aR [6–8], the terminal pathway components C6, C7 and C9, and terminal pathway regulators vitronectin and clusterin [9]. More recently, activated astrocytes have been shown to abundantly express and secrete C3 *ex vivo* and *in situ* in brain; indeed, C3 is now used as a marker for activated astrocytes [10]. Microglia, the principal brain phagocytes, are the predominant source of C1q in the brain [11]; cultured human primary microglia also expressed C3 [12] and induced pluripotent stem cell (iPSC)-derived microglia expressed C1q, C2, C3, complement receptor 3 (CR3) and complement receptor 1 (CR1/CD35) and several other complement proteins (Table 1) [13–15]. Neurons also express complement proteins; human neuronal cell lines expressed the components C1q, C2, C3, factor B (FB), C6 and C9, and the regulators factor H (FH) and C1 inhibitor (C1-INH) [16–18]. Human fetal neurons expressed C4, C3 and the regulators CD59 and CD46 and were positive for C3 and terminal pathway activation products, suggesting that complement was activated on them *in situ* [9]. Neurons derived from iPSC expressed and secreted C1, C4, FD, FI and FB as well as the regulators C1-INH, C1qBP and DAF [15,19]. Complement expression by the myelin-producing oligodendrocytes has been little studied; one report using a human oligodendrocyte cell line (HOG) showed expression of the membrane regulators CD55, CD46 and CD59 and the fluid-phase regulators C1-INH, vitronectin and clusterin [20,21]. RNA-sequencing analyses of individual brain cells have confirmed that most but not all complement genes are expressed in the human brain [22]. However, the majority of these studies were performed on non-inflamed brain; the release of transcriptomic data from the Seattle Alzheimer’s Disease Brain Cell Atlas should clarify brain cell expression of complement proteins in disease [23].

**Table 1:** Evidence of complement expression in different brain cell types

Protein	Neuron	Microglia	Astrocyte	Oligodendrocyte	References
**C1q**	Cell lines	iPSC, primary cells	Cell lines	Primary cells	[12,15,17,18,20,247,248]
**C1r**	iPSC	iPSC	Cell lines	Primary cells	[15,20,247–249]
**C1s**	iPSC	iPSC	Cell lines	Primary cells	[15,20,247–249]
**C2**	Cell lines	iPSC	Cell lines	Primary cells	[15,18,20,247–249]
**C3**	Cell lines, primary cells	Primary cells, iPSC	Cell lines, primary cells	Cell lines, primary cells	[9,12,15,17,18,20,247,250–253]
**C4**	Cell lines, iPSC, primary cells	Primary cells, iPSC	Cell lines, primary cells	Primary cells	[9,12,15 17,18,20,247–251,253–255]
**C5**	Cell lines, iPSC	iPSC	Cell lines	Primary cells	[15,17–20,247–249]
**C6**	Cell lines	iPSC	Cell lines, primary cells	Primary cells	[7,17,18,20,247,249]
**C7**	Cell lines	iPSC	Primary cells	Primary cells	[7,17,18,20,247]
**C8**	Cell lines	iPSC		Primary cells	[18,20,247]
**C9**	Cell lines, primary cells	iPSC	Cell lines, primary cells	Primary cells	[7,9,15,17,18,20,247,250,255]
**C1-INH**	Cell lines, iPSC, tissue	iPSC, primary cells	Cell lines, primary cells, tissue	Cell lines	[7,15,17,21,247,248,251,256,257]
**C3aR**	Cell lines	iPSC, primary cells	Cell lines, primary cells		[8,247,258–259]
**C5aR**		iPSC	Cell lines, primary cells		[6,15,247]
**C4BP**		iPSC		Cell lines	[21,247]
**C1qBP**	iPSC	iPSC			[15,19,247]
**MCP/CD46**	Cell lines, primary cells	iPSC	Cell lines, primary cells	Cell lines	[7,9,21,247,260–262]
**DAF/CD55**	iPSC	iPSC	Cell lines, primary cells	Cell lines	[9,19,21,247,259,261]
**CD59**	Cell lines, primary cells	iPSC	Cell lines, primary cells	Cell lines	[7,9,21,247,252,260,261]
**CFB**	Cell lines, iPSC	iPSC	Cell lines		[15,17,247,252]
**CFD**	iPSC	iPSC			[15,247]
**CFH**	Cell lines	iPSC	Cell lines, primary cells	Cell lines	[17,21,247,252,260,263]
**CFI**	iPSC		Cell lines		[15,252]
**Properdin**		iPSC	Primary cells		[15,247,263]
**CLU**	Cell lines, iPSC	iPSC	Cell lines, primary cells	Cell lines	[7,15,19,21,247,260]
**CR1**		Cell lines, iPSC	Cell lines, primary cells		[14,21,247]
**CR1L**		iPSC			[247]
**CR2**		iPSC			[247]
**CR4/CD11c**		iPSC			[15]
**CR3/CD11b/ITAM**		iPSC			[15]
**CSMD1**	Tissue				[204]
**FCN1**		iPSC			[247]
**FCN3**		iPSC			[247]
**Itgax**		iPSC			[247]
**MASP1**	iPSC	iPSC	Cell lines		[15,247,249]
**MASP2**	iPSC	iPSC	Cell lines		[15,247,249]
**MASP3**			Cell lines		[249]
**MBL2**		iPSC			[249]
**VTN**	Cell lines, iPSC	iPSC	Cell lines, primary cells	Cell lines	[7,15,21,247,260]

Abbreviation: iPSC, induced pluripotent stem cell.

Together, the data show that brain cells can make all the key complement proteins and regulators and express membrane complement regulators and receptors that enable them to resist and respond to complement activation (Table 1). Most complement proteins are acute phase reactants; peripheral inflammation drives increased hepatic synthesis with resultant increased plasma levels [24]. In brain, a few studies in primary cells, cell lines and mouse models have demonstrated that complement gene expression is increased by inflammatory triggers. Repeated systemic injection of lipopolysaccharide (LPS) in mice caused increased C1q expression in the hippocampus associated with increased microglial phagocytosis [25], while LPS treatment of neuron/glial co-cultures increased C3 expression and secretion [26]. The impact of inflammation on complement gene and protein expression in brain pathology will be discussed in later sections.

## Homeostatic and neuroprotective roles of complement in NDDs

CNS is an immune-privileged site isolated behind the BBB; complement proteins are locally synthesized to maintain immunosurveillance [27,28]. Protective roles include the clearance of pathogens and cellular debris, synaptic pruning and neural circuit refinement, and maintaining BBB integrity. C1q has anti-inflammatory and neuroprotective properties, accomplished by enhancing the removal of apoptotic cells, suppressing inflammatory cytokines, encouraging the quiet elimination of cell debris, and reducing the accompanying inflammation [29,30]. Additionally, throughout retinal development, C1q with C3 contribute to pruning and remodeling of synapses [31]. The local activation of C3 to C3b, which can subsequently function as an opsonin, occurs at synapses that are C1q tagged. This enables microglia expressing the C3b receptor, CR3, to specifically target and eliminate these neurons. C1q and C3 have also been linked to memory regulation, specifically in the process of forgetting, where they direct microglial engulfment of specific engram cells during restructuring of the brain [32]. Downstream complement pathway activation leads to neurotoxicity [28,33]. Studies utilizing animal models of AD, stroke and NMO demonstrate the significance of complement in maintaining the integrity of the BBB and the healthy balance of the brain, although the precise protective mechanisms are uncertain [34]. Together, these data show that complement is critical for immunosurveillance and essential for normal brain development. However, chronic activation of the cascade contributes to the breakdown of the BBB and brain injury.

## Complement at the synapse

The demonstration that complement is required for synaptic pruning during development was a game-changer, focussing attention on roles of complement in brain health and disease [31,35]. These elegant studies in the developing rodent visual system showed that synapses destined for elimination were tagged with C1q [31]. Later studies showed that synaptic C1q is part of the C1 complex, and that binding of C1 initiated activation of the classical complement pathway on the targeted synapse, leading to the deposition of C3 activation fragments (C3b and iC3b) and synaptic engulfment by microglia expressing C3 fragment receptors (CR3 and CR4) [36,37]. Mice deficient in C1q, C3, C4 or the receptor CR3, all showed defects in developmental synaptic pruning [35–38]. Together, these studies demonstrated a critical homeostatic role for complement in synapse elimination and confirmed that complement was present and active in the healthy brain, provoking further research activity.

**Figure 2:**
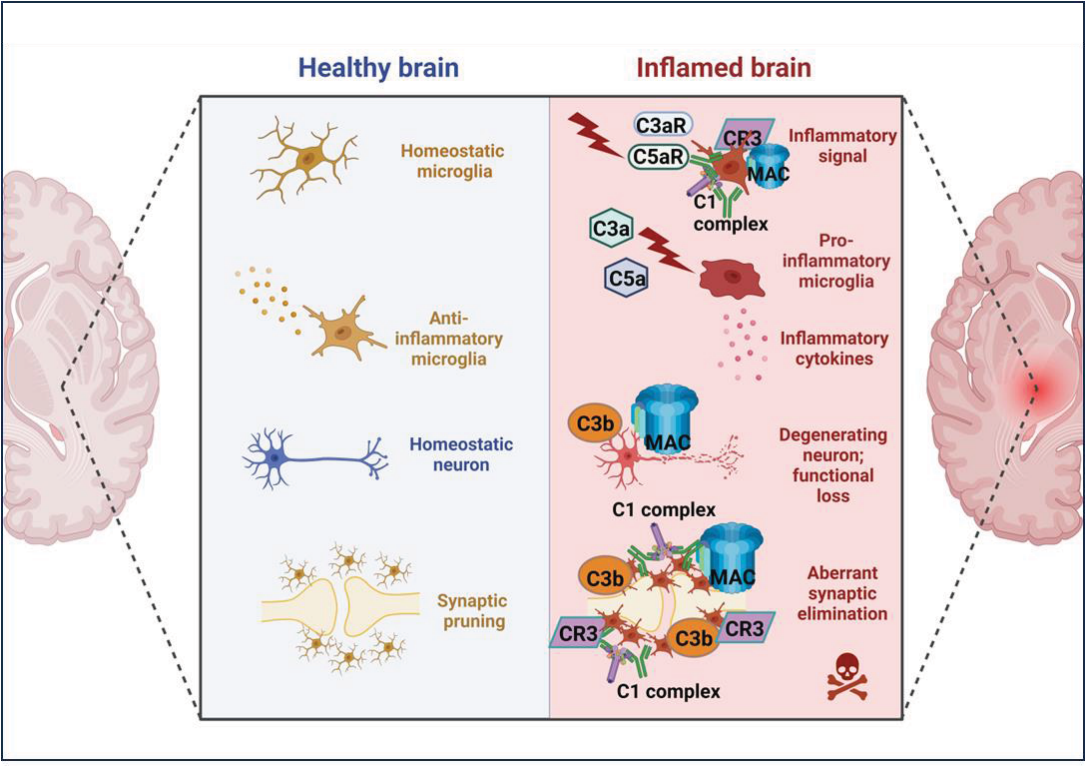
Complement in healthy and neurodegenerative brain In the healthy brain, as in other organs, complement plays a crucial role in defending against infections and maintaining tissue homeostasis. These homeostatic roles include the core complement functions of immune surveillance to detect and eliminate pathogens and to remove dead cells and debris (in collaboration with microglia), and the brain-specific role of synaptic pruning. In the NDD brain, complement is dysregulated, generating an excess of pro-inflammatory products that trigger hyper-activation of microglia (and other glia), production of diverse proinflammatory mediators and increased phagocytosis. Terminal pathway activation and MAC formation directly damages neurones and synapses, precipitating neurodegeneration. Figure created with BioRender (BioRender.com).

In the following sections, we will discuss the evidence implicating complement in the various neurodegenerative diseases (NDDs) and strategies to modulate complement activation in brain. Likely roles of inflammation and complement dysregulation in NDDs are summarized in Figure 2.

## Complement roles in Alzheimer’s disease

Alzheimer’s disease (AD) is the commonest cause of dementia, a NDD affecting more than 55 million individuals worldwide and projected to increase dramatically in the next decade as the population ages [39,40]. AD causes a progressive deterioration in cognitive abilities, memory, and ability to perform everyday tasks, ultimately resulting in a loss of independence. The pathological hallmarks of AD are the accumulation in brain of protein deposits, amyloid-β (Aβ) plaques and hyperphosphorylated tau tangles. Cognitive impairment is caused by loss of neurons and synapses in specific brain regions. Neurodegeneration is accompanied by inflammation; brain-resident immune cells, astrocytes and microglia, adopt a neurotoxic, phagocytic, and proinflammatory phenotype and interact with plaques, tangles and damaged neurons [41,42]. This neuroinflammation, once dismissed as a secondary event, is now recognized as a significant driver of AD pathology. The most compelling evidence came from genetic studies; several of the genes most strongly associated with AD risk are related to inflammation and immunity [43–45]. The realization that neuroinflammation is causative in AD has refocussed research to gain a deeper understanding of the timing, location and mechanisms of inflammation in the AD brain, identify the inflammatory pathways, develop improved diagnostic tests and design innovative therapeutic interventions.

### Complement in AD: immunohistochemistry (IHC) evidence

While restricted to postmortem AD brain and thus to late-stage disease, IHC analysis has nevertheless convincingly demonstrated the presence of complement proteins and activation products in and around areas of pathology. Classical pathway proteins, including C1q, C3 and C4, co-localized specifically with amyloid plaques but not tangles in temporal cortex, amygdala, and hippocampus, the brain regions most affected in AD [2,46,47]. Complement activation products, including C3 fragments and the terminal complement complex (TCC), were abundant in AD cortex, associating with plaques, tangles, and neuropil threads [48]. Microglia proximal to areas of pathology in the hippocampus and frontal cortex showed up-regulation of C5a receptors, C5aR1, and C5aR2 [49], while microglial CR1 expression was increased 10-fold in AD cases compared with controls [14].

### Complement in AD: genetic evidence

Multiple genome-wide association studies (GWAS) have identified single-nucleotide polymorphisms in complement genes as contributing factors to late onset AD (LOAD) risk [45,50–52]. Two complement genes are reproducibly identified in the top five genetic hits: *CR1* encoding CR1, an important receptor and regulator of complement activation, and *CLU* encoding clusterin, a chaperone protein involved in several homeostatic processes, including complement regulation [50,51,53]. The demonstration by microarray analysis that clusterin expression was upregulated in AD brain supported the association [54]. A recent GWAS additionally identified *C1S*, encoding the key C1-associated enzyme C1s, as a potential genetic factor in LOAD, firmly implicating the classical pathway in AD pathogenesis [50]. Other AD GWAS hits include *CD33* and *TREM2*, both encoding myeloid cell receptors that bind C1q and/or C3 [55,56]. Expression studies have reported elevated expression of the complement receptors C3aR1 and C5aR1 and complement proteins C4A, C4B and CFHR1 in AD compared with age-matched controls, while C1q binding protein (C1qBP) expression was decreased [54]. In a separate study, the macular degeneration-associated risk polymorphism in FH (1277C) was associated with higher risk of AD [57]. While these genetic associations provide strong evidence of the involvement of complement in AD pathology, the precise mechanisms by which complement gene variants influence AD risk remain largely unknown. Research in this area is ongoing, aiming to unravel the complex interplay between genetic factors and complement dysregulation in AD development, essential to inform future therapeutic strategies and interventions.

### Complement in AD: biomarker evidence

Numerous studies have identified distinct alterations in complement proteins and activation products in blood and/or CSF from AD patients compared with controls, or as predictors of progression to AD in individuals with mild cognitive impairment (MCI) (Table 2) [58–62]. These have been comprehensively reviewed elsewhere [63] and will be summarized briefly here. In CSF, several studies have reported increased levels of C3 and FH in AD, correlating with cognitive decline [64–66]; however, another study reported that lower levels of C3 and FH were associated with faster cognitive decline and increased brain atrophy [67]. Others reported increased C4 and soluble CR1 in AD CSF [68]. In plasma, low C3 levels were associated with risk of progression to AD, particularly in those with apolipoprotein E4 (APOE4) genotype [62]. Individuals with Down Syndrome (DS) and dementia had significantly lower plasma levels of C3 and factor I compared with those without dementia [69]. A recent study linking genetics and plasma complement levels demonstrated that AD-associated SNPs in *CR1*, *C1S* and *FH* impacted plasma levels of the encoded proteins, suggesting a mechanism for the observed impact on disease risk [70]. Although there is a lack of consistency between these many studies, likely influenced by different populations and very different assays, collectively they support altered complement activity and regulation in AD patients.

**Table 2:** Complement biomarkers in neurodegenerative diseases

Disease	Sample type	Complement component	Change	Reference
**Alzheimer’s disease**	Plasma	C3	Decrease	[62]
	CSF	C3, FH	Increased	[64–66]
	CSF	C3, FH	Decrease	[67]
	CSF	C4, CR1	Increased	[68]
**Down syndrome**	Plasma	C3, FI	Decrease	[69]
**Frontotemporal dementia**	Plasma	C2, C3	Increased in C9orf72 and MAPT carriers	[112]
	CSF	C1q, C3b	Increased	[112]
	CSF	C2, C3, FD	Increased	[112]
**Multiple systems atrophy**	Plasma	C3	Decrease	[65,66]
	CSF	C3	Decreased	[65,66]
**Parkinson’s disease**	Plasma	C3	Decrease	[65]
	Plasma	C3	No change	[120]
	Plasma	C3, C4	Increased baseline levels	[120,121]
	CSF	FH, C9	Increased	[123]
**Huntington’s disease**	Plasma	C7, C9, Clusterin	Increased	[141]
	CSF	C1q, C4B	Increased	[141]
**Multiple sclerosis**	Plasma	MASP-2, C3, C4, C4a, FH, C1inh	Increased	[150,160–162,171]
	Plasma	iC3b, Bb, C1inh, C1s, C9, TCC	Decreased	[160,163]
	Plasma	C4, FB, Bb, C1s, Clusterin, FI	No difference	[150,160,162]
	CSF	C1q, C3, C3a, C4, C4a, TCC	Increased	[150,165,166,168,169,171]
	CSF	C3, C9, FB	Decreased	[160,166,167]
	CSF	C3, C4	No difference	[162]
**Schizophrenia**	Plasma	C4a	Increased	[218]

### Complement in AD: evidence from animal models

There have been numerous studies of the impact of complement component knockout or complement inhibition on disease in mouse models of amyloid and tau pathology with rather mixed results. The demonstration that Aβ oligomers directly injected into mouse brain triggered complement activation and synapse loss, prevented by administration of C1q blocking antibody, implicated complement in amyloid-induced pathology [71]. In multiple mouse AD models, knockdown of complement proteins protects synapses [72]. For example, C1q knockout reduced synaptic pruning by both astrocytes and microglia and rescued synaptic density [73], and anti-C1q treatment rescued synapse loss in the P301S mouse model [74]. Knockout of the pivotal complement protein C3 protected synapses and neurons in the APP/PS1 mouse AD model despite increased plaque load, demonstrating a disconnect between amyloid plaque accumulation and neurodegeneration [75]. Nevertheless, the absence of C3 in the APP-C3^−/−^ mice resulted in an accelerated deposition of amyloid beta plaques and increased neurodegeneration [76]. Moreover, 1-year-old APP mice expressing the C3 inhibitor soluble complement receptor-related protein y (sCrry) exhibited a 2- to 3-fold higher amyloid beta deposition accompanied by accumulation of degenerating neurons when compared with age-matched APP mice [77]. This dichotomy highlights the intricate interplay between complement and amyloid beta pathology, underscoring the need for a nuanced understanding of their relationship in the context of neurodegenerative processes. The terminal pathway, responsible for MAC formation, has also been implicated in models; knocking out C6 in 3xTg AD mice reduced synapse loss and pharmacological blockade of the terminal pathway using an anti-C7 blocking antibody was neuroprotective in APP^NLGF^ mice, together indicating that MAC formation plays a significant role in driving synapse loss in AD models [78]. The C3a/C3aR pathway has been implicated in numerous animal studies. *Helicobacter pylori*-derived outer membrane vesicles caused accelerated disease development in a mouse AD model and this was reversed by disrupting C3a/C3aR signaling [79]. Knockout of C3aR reduced microglial activation, synapse loss and cognitive decline in both tauopathy [80] and amyloidopathy [81,82] models. The C5a/C5aR pathway has also been implicated; both C5aR1-KO and inhibition of the C5a/C5aR pathway restored microglial homeostasis, reduced neuronal loss and improved cognition in AD models [83,84].

### Complement in AD: summary

These combined findings in human AD samples and AD models highlight the involvement of diverse components of the complement system in pathogenesis and provide valuable insights into the likely roles of complement in the AD brain. A better understanding of complement dysregulation in early disease stages and progression would aid development of effective diagnostic and therapeutic strategies for AD.

## Complement roles in vascular dementia

Vascular dementia (VaD) is the second most common cause of dementia and a frequent comorbidity in AD and other neurogenerative diseases, present in >50% of dementia cases [85]. In VaD, blocked or reduced blood flow to the brain leads to reduced brain oxygen and glucose, essential for neuronal survival. Underlying pathologies include atherosclerosis, small vessel disease (SVD) and cerebral amyloid angiopathy (CAA); all can lead to cerebrovascular infarcts, intracerebral haemorrhage (ICH) and chronic cerebral hypoperfusion. Neuroinflammation is an integral part of VaD and increasing evidence demonstrates the importance of complement in its pathology.

### Complement in VaD: contributions to risk factors

Risk factors for VaD include stroke, diabetes and hypertension, all of which are associated with atherosclerosis [85]. Complement dysregulation is strongly implicated in atherosclerosis as a driver of inflammation and so likely plays a role in SVD and VaD [86]. Early studies reported deposition of complement proteins, including the activation product iC3b and regulator FH, in atherosclerotic plaques [87]. In two large GWAS, increased risk of ICH was strongly associated with genetic variation in the complement genes *CR1* and *Clu*, both also AD GWAS significant [88], implying a causal role of complement in VaD pathogenesis. In a mouse model of ICH, neurodegeneration and edema were reduced in C3-KO mice [89], while pharmacological inhibition of complement with N-acetylheparin or tricarboxylic acid reduced brain injury in a rat ICH model [90]. These findings implicate complement in the pathogenesis of VaD induced by microinfarcts and suggest that complement inhibition might ameliorate injury.

### Complement in VaD: roles in CAA

CAA, an accumulation of amyloid in the walls of small cerebral arteries, is present to varying degrees in ∼90% of AD cases, particularly those with underlying SVD [91]. Impaired clearance of amyloid along intramural periarterial drainage (IPAD) pathways is implicated in the development and progression of CAA [92]. Vascular amyloid deposits are associated with damage to the endothelium and perivascular inflammation resulting in vasogenic oedema observed as white matter hyperintensities (WMHI) in MRI [92–94]. The recent demonstration that plasma C3 levels are elevated and a potential blood biomarker of CAA in MCI patients suggests a role for complement [95]; indeed, an IHC study of CAA in AD brain showed deposition of C3 fragments and C9 in the amyloid-laden vessels that correlated with severity of CAA [96], while others report staining for C1q, C3d, C6 and C5b-9 in amyloid-positive cerebral vessels from CAA cases [91]. Brain vascular smooth muscle cells expressed all classical and terminal pathway complement proteins, increased on exposure to Aβ, IFN-γ or IL1β, suggesting that local production might contribute to complement dysregulation in the vessel wall [97]. Proteomic studies in white matter or isolated amyloid-laden blood vessels from CAA donors showed enrichment in the terminal pathway regulator clusterin, suggesting a compensatory mechanism to limit MAC formation [98,99]; in contrast, genetic ablation of clusterin in AD mice reduced amyloid load and brain inflammation by causing redistribution of amyloid from plaques to the perivascular compartment [100]. Of note, immune complex formation and complement activation within IPAD pathways is a frequent complication in trials of anti-amyloid immunotherapy and a major driver of toxic side effects including microhaemorrhages [101]; inhibiting complement activation could alleviate the toxicity.

### Complement in VaD: roles of C3a/C3aR

The C3a/C3aR pathway is an important contributor to vascular inflammation. Human brain microvascular endothelial cells stained *in situ* or in culture, expressed C3aR; exposure to C3a increased BBB permeability through disruption of tight junctions and enhanced perivascular inflammation via VCAM up-regulation [102]. Hypoperfusion, a common characteristic of VaD, caused microglial activation in mice with up-regulation of C3 and C3 fragment receptors, C3aR and CR3 and resultant demyelination with complement activation and C3 fragment deposition on myelin [103]. Genetic ablation or pharmacological inhibition of C3aR reduced hypoperfusion-induced microglial activation and prevented myelin loss, confirming the primary role of the C3a/C3aR axis in hypoperfusion injury and its potential as a target for therapeutic intervention in VaD [103].

### Complement in VaD: summary

Studies in man and models convincingly demonstrate the relevance of complement dysregulation in the conditions that predispose to VaD, notably vascular diseases and stroke. Dysregulation of complement in the vessel walls, particularly in the context of CAA, is likely an important exacerbating factor and trigger for vascular inflammation in VaD. Targeted complement therapies might benefit patients by impacting complement dysregulation.

## Complement roles in frontotemporal dementia with tau pathology

Frontotemporal dementia (FTD) describes a group of diseases characterized by degeneration of neuronal circuits in frontal and temporal lobes. FTDs are pathologically diverse, presenting with proteinaceous inclusions of tau in FTD-tau, transactive response DNA-binding protein (TDP-43) aggregates in FTD-TDP, or fused in sarcoma (FUS) aggregates in FTD-FUS [104]. FTD is a largely heritable disorder with 10–20% of cases reporting autosomal dominant mutations within microtubule associated protein tau (MAPT), progranulin (GRN) or chromosome 9 open reading frame 72 (C9orf72) [104]. The most common form of sporadic FTD is FTD-tau; this term includes progressive supranuclear palsy (PSP), corticobasal degeneration (CBD), Pick’s disease (PiD), argyrophilic grain disease (AGD), globular glial tauopathy (GGT), age related tau astrogliopathy (ARTAG) and primary age-related tauopathy (PART) [105].

Although GWAS have revealed strong associations with microglia function and inflammatory processes in FTD-tau, a direct genetic association with complement has not been identified [105–107]. The demonstration that Tau aggregates, when tested *in vitro*, potently and directly activate classical complement pathway in an antibody independent manner [108], together with mounting evidence implicating complement from postmortem and animal model studies, suggests that complement dysregulation contributes to the underlying pathology.

### Complement in FTD-tau: IHC evidence

Direct evidence for complement dysregulation in the brain in FTD is scarce. Early IHC analyses in postmortem brain tissue identified complement components and activation products, predominantly from the classical and terminal pathways, localized to reactive astrocytes and neurons containing Pick bodies in cases of PiD [109], and on neurofibrillary tangles in cases of AD with tau pathology [110] and Guam Parkinsonism-dementia [111]. More recent evidence for complement association with tau pathology comes from AD, which is considered a secondary tauopathy, although complement is mainly localized to plaques, not tangles [2], likely because tau accumulation is mainly intracellular only released after cell death. Nevertheless, these findings suggest that tau aggregates can activate complement and downstream events in the brain.

### Complement in FTD-tau: biomarker evidence

In contrast to AD where complement biomarkers are well studied, there is a paucity of published studies of complement levels in FTD (Table 2). A recent report from the Genetic Frontotemporal Dementia Initiative (GENFI) used multiplexed assays to measure CSF and plasma levels of complement components and activation products in FTD cases with known genetic mutations in *MAPT*, *C9ORF* or *GRN* at different stages of the disease [112]. C1q and C3b were elevated in CSF during the symptomatic stage compared with presymptomatic carriers and non-carriers. The increased C1q and C3b levels correlated with CSF Nfl levels and decreased brain volume, indicative of cerebral atrophy and neurodegeneration [112]. In the symptomatic stage, C2, C3 and FD also correlated with reduced whole brain volume, further implicating complement in disease progression [112]. In plasma, C2 and C3 were elevated in FTD linked to *C9orf72* and *MAPT*, but not *GRN* [112].

### Complement in FTD-tau: evidence from animal models

Studies in mice harboring the P301S/L tau mutation have informed understanding of complement dysregulation in response to tau pathology [113]. Neuronal accumulation of mutant tau triggered classical complement gene expression by microglia and astrocytes and consequent neurodegeneration [74]; C1q was enriched at the synapse correlating with tau pathology, and provoking the suggestion that C1q-initiated synaptic loss causes neurodegeneration in this model. Indeed, knockout of C1q protected the P301S mice from synapse and neuronal loss and brain atrophy [73,113]. Knockout of C3 or C3 inhibition in brain by overexpression of the C3 inhibitor sCrry conferred similar protection in the mouse model, further implicating complement dysregulation [114]. sCrry over-expression also reduced tau pathology in P301L mice, suggesting that complement dysregulation contributes to tau aggregation [114]. In marked contrast, lentiviral silencing of Crry in P301S mice reduced tau phosphorylation and neuronal loss with improvement in behavioral outcomes [115]; these authors suggested that increased C3 turnover and C3b generation in the absence of Crry enhanced clearance of neurotoxic tau, illustrating the complex, double-edged nature of complement.

Involvement of the terminal pathway in tau pathology was also explored in P301L mice. Knockout of the cell-bound MAC inhibitor CD59 caused increased tau phosphorylation in the mice, likely due to MAC-induced cell activation, accompanied by increased synapse and neuronal loss [114]. C3aR expression on microglia was markedly increased in PS19 mice compared with controls, and C3aR KO reversed vascular abnormalities, reduced gliosis, tau hyperphosphorylation and synapse loss, and protected hippocampal dependent memory and LTP [80,102]. Pharmacologic blockade of C3aR in P301S mice impacted p35/CDK5 signaling in microglia, reduced synapse loss and tau hyperphosphorylation, and improved spatial memory [116]. Contributions of the C5a/C5aR axis were tested using the C5aR antagonist PMX205 in the 3xTG AD model (includes the P301L tau mutation); treatment for 3 months reduced tau hyperphosphorylation and rescued cognitive function compared with controls [117]. These latter studies suggest that drugs targeting C3a/C3aR and/or C5a/C5aR pathways may ameliorate disease in FTD-tau.

### Complement in FTD-tau: summary

Despite the scarcity of research on complement in human FTD cases, the studies reported here demonstrate a clear involvement of complement dysregulation. The role of complement in tauopathies resembles that in AD, particularly roles in synapse loss. The impact of complement inhibition in mouse models of FTD-tau provides a strong rationale for further studies to inform future development of complement therapeutics in FTD.

## Complement roles in synucleinopathies

Synucleinopathies comprise a group of NDDs characterized by accumulation of α-synuclein (αSyn), predominantly in the form of Lewy bodies (LB) that accumulate in dopaminergic (DA) neurons and/or glial cells. Synucleinopathies are broadly divided into two disease groups, the LB diseases Parkinson’s disease (PD) and Dementia with LBs (DLB), and the Multiple systems atrophy (MSA) group, itself divided into MSA with predominant cerebellar ataxia and MSA with predominant parkinsonism [118]. Why and how defects in αSyn processing cause different types of aggregates and different pathologies remains unclear. Regardless, αSyn accumulation leads to the progressive loss of neurons in the basal ganglia of patients [119]. Reactive microglia are abundant in areas of neuronal loss and GWAS have implicated inflammation and immunity pathways, implying that neuroinflammation is a core feature [119].

### Complement in synucleinopathies: biomarker evidence

A few studies have investigated CSF or blood complement levels in synucleinopathies with varying results (Table 2). C3 levels were consistently decreased compared with controls in both CSF and serum from MSA but not PD [65,66]. Others reported decreased plasma C3 levels [65], or no difference in PD compared with controls [120], while high baseline levels of C3 and C4 predicted worse outcome in a longitudinal biomarker study in PD [120,121]. A proteomic analysis of PD CSF identified the alternative pathway regulator FH as one of the most significant differentially altered proteins [122]. A recent large proteomic study in over 1000 serum samples from PD and healthy controls identified enrichment of complement proteins; C9 was strongly associated with disease status, cognitive decline and motor symptoms implicating the terminal pathway [123]. Analysis of complement activation products is still largely lacking in these studies and would clarify the extent of complement dysregulation in synucleinopathies.

### Complement in synucleinopathies: IHC evidence

Several IHC studies of postmortem brain from PD and DLB cases have demonstrated association of complement proteins and activation products with the hallmark lesions. C1q and the activation markers iC3b, C3d and C4d all localized to LBs in dopaminergic neurons in the substantia nigra, but AP-specific markers were absent, implicating classical pathway activation [124–128]. Terminal pathway components C7 and C9 were also localized to the lesions, implying activation through to MAC. Activated microglia were closely opposed to C1q/C4d-positive LB-bearing dopaminergic neurons, suggesting that phagocytic clearance of damaged neurons was underway [124,125]. In support of these findings, the microglial receptors for C3 activation products, CR3 and CR4, were markedly up-regulated in RNAseq analysis of cortex from 29 PD cases [129,130].

### Complement in synucleinopathies: evidence from *in vitro* studies and animal models

αSyn is a 140 amino acid protein with an amphipathic lysine-rich amino terminus involved in interactions with membranes and a carboxy-terminal region implicated in nuclear localization. Direct activation of complement by αSyn, either expressed on a neuronal cell line or as isolated protein, has been demonstrated *in vitro*. Fibrillar αSyn incubated with serum bound C1 via the carboxy-terminus activated the classical pathway and became coated with C4b [131–133]; microglia selectively bound this opsonized fibrillar αSyn through the phagocytic receptors CR3 and CR4 to mediate clearance.

A few studies have investigated roles of complement in PD caused by exposure to pesticides and toxins that cause dopaminergic neuron loss. In mice, repeated dosing of rotenone caused neuronal loss accompanied by up-regulated microglial CR3 expression; knockout of CR3 prevented the neurodegeneration, suggesting a primary role for complement [134]; however, prevention of complement activation by knocking out C1q or C3 did not reduce neurodegeneration in another toxin-induced PD model, again demonstrating the inconsistencies between studies and models [135,136].

### Complement in synucleinopathies: summary

Taken together, these studies implicate complement dysregulation in some types of synucleinopathies; complement is activated by αSyn aggregates via the classical pathway, may facilitate removal of aggregates via opsonization, but also enhances inflammation, a double-edged sword. To date, no trials of anti-complement drugs in this group of diseases have been reported.

## Complement roles in Huntington’s disease

Huntington’s disease (HD) is an inherited NDD characterized by a choreatic movement disorder with psychiatric symptoms, progressive neurodegeneration and dementia. The underlying cause is an expansion of a CAG triplet repeat in exon 1 of the *HTT* gene encoding the ∼350 kDa protein huntingtin. This generates an elongated polyglutamine tract that causes mis-folding, aggregation and accumulation of protein in neurons and other cells in the striatum and cortex leading to neuronal loss, gliosis and brain atrophy [137]. Neuroinflammation is a feature of the disease but has been considered a secondary event and attracted limited attention [138].

### Complement in Huntington’s disease: evidence from IHC studies

IHC in postmortem brain tissue from HD cases has demonstrated complement dysregulation specifically in areas of pathology. Neurons, astrocytes and myelin sheaths in the caudate and striatum of HD patients showed staining for the complement proteins C1q, C4 and C3, and the activation products iC3b and TCC, strongly indicating complement dysregulation [139]. Astrocytes and microglia up-regulated C5aR expression in the caudate nucleus of HD patients [140].

### Complement in Huntington’s disease: evidence from biomarker studies

There have been very few studies measuring complement proteins in HD blood and/or CSF; plasma proteomic profiling identified increased levels of C7, C9 and clusterin, the latter also increased in CSF [141], although another study failed to replicate these changes in HD plasma, perhaps due to differences in assays or patient cohorts [142]. CSF levels of C1q and C4b were strongly associated with disease severity and predictive of rate of progression in premanifest HD mutation carriers [143]. No studies of complement activation products in HD are reported.

### Complement in Huntington’s disease: evidence from *in vitro* studies and animal models

Studies of complement in mouse HD models have predominantly focused on the R6/2 model, expressing human *HTT* exon 1 with 150 CAG repeats under the human huntingtin promoter [144]. R6/2 transgenic mice display progressive neurological and behavioral features resembling HD. Complement proteins were not up-regulated in the mice and crossing to C3 deficiency had no impact on HD disease progression [145]. In a chemically induced rat model of HD (3-nitropropionic acid, 3-NP) C3, C9 and C5a receptor (C5aR1 and C5aR2) expression was increased in striatal neurons in regions of pathology [146]. Administration of C5aR antagonists (PMX53 and PMX205) either pre- or post-3-NP induction reduced HD phenotypes; including weight loss and motor deficits such as ataxia and dystonia [146], suggesting a role for complement-driven inflammation in the disease process.

### Complement in Huntington’s disease: summary

There is limited but growing evidence that complement is dysregulated in HD while animal models implicate inflammation and complement in pathogenesis. The limited data have provoked testing of anti-complement therapeutics in AD. Annexon has completed a Phase 2 study of their anti-C1q mAb in HD with promising results in a subset of patients displaying evidence of complement dysregulation [147]; expansion to Phase 3 is planned in 2024.

## Complement roles in multiple sclerosis

Multiple sclerosis (MS) is a complex neuroinflammatory disease characterized by demyelination and neurodegeneration. While the primary cause of MS remains unknown, neuroinflammation plays key roles in demyelination, neurodegeneration, and disease progression [148–159]. Originally considered a disease of white matter (WM), it is now clear that demyelinating lesions are found globally in the MS brain. Current therapies target adaptive immunity and are effective in many, but not all cases.

### Complement in MS: biomarker evidence

There have been numerous studies measuring complement biomarkers in MS plasma and/or CSF (Table 2); however, reported results are variable and contradictory, likely because of a lack of standardized assays. Significant differences have been reported for plasma levels of components (C1s, MASP-2, C3, C4 and C9), regulators (FH and C1inh) and activation products (iC3b, Bb, C4a, C4b and TCC) between MS patients and controls and between MS patients in remission, relapse or with progressive disease [150,160–164]. A recent study reported increased CSF levels of C1q and C3a in newly diagnosed patients with clinically isolated syndrome and RRMS compared with healthy controls, which correlated with several inflammatory chemokines, implicating complement dysregulation in early disease [164–171].

### Complement in MS: IHC evidence

MS is pathologically heterogeneous. Lassmann and co-workers classified cases based on pathology into four groups (I–IV) and showed that complement deposition in demyelinating lesions was a defining feature in many cases, including most of those with primary progressive MS (ppMS) [172,173]. Complement components, regulators and activation products all localize to lesions; the presence of the latter, C3 fragments and terminal pathway complexes, confirm complement dysregulation in the lesions [148,158]. In myelin oligodendrocyte glycoprotein (MOG) antibody associated disorders (MOGAD), an MS-related condition defined by the presence of anti-MOG antibodies, all active demyelinating plaques were strongly positive for complement activation products [174].

Complement activation products have also been demonstrated in the leptomeninges associated with underlying cortical demyelination, inflammation, and microglial activation in MS brain [159]. Recently, complement activation products were found in abundance in thalamic grey matter lesion in ppMS, implicating complement as a driver of grey matter pathology [156]. Taken together, the IHC data suggest an ‘outside-in’ gradient of complement dysregulation that causes further barrier disruption and increased pathology, reviewed elsewhere [175]. Overall, studies in human postmortem brain demonstrate that complement is dysregulated in a large proportion of MS cases, including most of those with ppMS.

### Complement in MS: evidence from *in vitro* and animal models

Experimental autoimmune encephalomyelitis (EAE) is the benchmark mouse model of MS; roles of complement have been explored in the model either by testing the effect of complement gene knockouts or using anti-complement drugs. Systemic delivery of the C3 blocker sCR1 inhibited demyelination and ameliorated paralysis and weight loss in acute EAE [176]; however, intraparenchymal delivery of another C3 inhibitor, Crry, decreased synaptic loss but did not impact demyelination [177]. In knockout mice, C3 KO was protective against microglial activation and synapse loss in the hippocampus and reduced the severity of motor deficits; in contrast, C1qa KO showed no significant effect [178]. The terminal pathway was implicated in demyelination in EAE in two studies; deficiency of C6 in rats protected against demyelination and paralysis whereas knockout of the terminal pathway inhibitor CD59 in mice exacerbated demyelination and symptoms [179,180].

### Complement in MS: summary

There is overwhelming evidence from EAE and human studies demonstrating the utility of complement as a biomarker of disease severity, to distinguish between the different MS subtypes, and as a marker of pathology. Evidence of complement involvement in the pathogenesis of MS, at least in some subtypes of the disease, continues to emerge as more studies implicate locally generated and circulating complement and demonstrate complement dysregulation centrally and peripherally. The evidence from EAE models supports a critical role for complement in demyelination, likely involving both opsonization of myelin to facilitate phagocytic removal and terminal pathway-mediated damage impacting myelin and axons. Although more research is needed to understand the role of complement dysregulation in MS, its impact at different stages of the disease and precisely which complement effectors are responsible for myelin loss, axonal injury, synapse loss and neuronal damage, trials of anti-complement drugs in those with proven complement dysregulation are long overdue.

## Complement roles in schizophrenia

Schizophrenia is a chronic and debilitating neuropsychiatric disorder that affects approximately 0.7% of the population [181]. Symptoms include delusions, hallucinations, disorganized speech and negative symptoms (e.g., anhedonia); the disorder is associated with an increased rate of mortality especially due to suicide and smoking-related diseases [182,183]. Postmortem studies reveal reduced cortical synaptic density, cortical thickness, and grey matter volume, consistent with neurodegeneration [184–187], supporting suggestions that defective cortical synaptic pruning underlies the condition [188]. In the normal developing brain, an excess of synaptic connections are generated that are removed during maturation of neural circuits; in schizophrenia this latter process is impaired [189,190]. Complement is established as a driver of developmental synaptic pruning [31,35] and evidence implicating complement dysregulation in the pathogenesis of schizophrenia is accumulating.

### Complement in schizophrenia: genetic evidence

GWAS have shown that schizophrenia is highly polygenic; indeed, twin studies show that ∼80% of variance in schizophrenia susceptibility is inherited [191–193]. The first evidence of a link to complement came from the demonstration that the major GWAS association in the major histocompatibility complex (MHC) on chromosome 6 was in the *C4* locus [38,193–195]. This locus comprises two alleles, *C4A* and *C4B*, encoding C4A and C4B respectively; these isoforms differ in just a few amino acids but are functionally distinct, the C4A thioester preferentially binding amino groups while C4B thioester preferentially binds hydroxyl groups [196,197]. *C4* copy number varies and schizophrenia risk is increased in proportion to both the number of *C4A* alleles present and the expression level of *C4A* in the brain, leading to the suggestion that C4A more efficiently labels synapses to drive increased complement-mediated synaptic pruning [38]. More recent GWAS have identified additional risk-associated loci including CUB and sushi multiple domains 1 (*CSMD1*) [198–200]. *CSMD1* encodes a large transmembrane receptor predominately expressed in brain and reproductive tissues, reported to be a complement inhibitor [201–204]. Precisely how *CSMD1* variants predispose to schizophrenia is unknown. Reduced PBMC expression of *CSMD1* in people with schizophrenia was reported in a small Han Chinese cohort [205]. Another study replicated the finding of reduced *CSMD1* expression in PBMC and correlated this with reduced CSMD1 levels in plasma [206]; however, as CSMD1 has no reported secreted form, the source of CSMD1 in plasma is unclear. Recently, reduced *CSMD1* expression was also reported in first episode psychosis PBMCs [207].

SNPs at the *CSMD1* locus have been linked to several other neurological disorders, including Parkinson’s disease [208], bipolar disorder [209], multiple sclerosis [210,211], autism and learning disorders [212,213], implying that *CSMD1* has a broad role in brain development and function, perhaps linked to synaptic loss, a common feature of these disorders. CSMD1 expressed at the synapse may regulate complement activation and synaptic opsonization locally, restricting microglial elimination [31,35,38].

### Complement in schizophrenia: evidence from animal models

Several mouse studies have explored the impact of C4 on schizophrenia, although they are complicated by the fact that mice only have one *C4* gene; mice humanised to express *C4A* and *C4B* have been made and used to show that C4A expression caused excessive synaptic pruning in the developing and adult mouse brain compared with C4B [214]. Others reported that *C4A* overexpression in mice induced enhanced microglial synaptic removal and schizophrenia-like behaviors [214,215].

### Complement in schizophrenia: evidence from biomarker studies

Complement component levels and mRNA expression in plasma and brain have been repeatedly investigated as candidate biomarkers of schizophrenia and the results thoroughly reviewed elsewhere [216]. Results are inconsistent although multivariate models including multiple complement analytes measured in plasma show some promise [217]. A recent proteomic study reported elevated plasma C4A levels in schizophrenia cases, linking with the genetics and implying that measurement of the C4A isotype may provide a more suitable biomarker than measurement of total C4 [218].

### Complement in schizophrenia: summary

The critical role of complement in developmental synaptic pruning and the strong genetic evidence implicating complement proteins and regulators together provide significant evidence implicating complement dysregulation in schizophrenia. Further work to better define roles of complement in synaptic pruning and to select the best markers of complement dysregulation, particularly in the early stages of the disease, are needed to identify whether and when complement dysregulation occurs in schizophrenia.

## Complement drugs in NDDs

The sections above implicate complement dysregulation in diverse NDDs. Numerous studies using anti-complement drugs in animal models of NDDs have provided proof of concept for complement inhibition, summarized in Table 3. Here we will briefly discuss the evolution of anti-complement drug therapy of NDDs. Several of the existing toolbox of anti-complement drugs are already in the clinic or in clinical trials in a handful of NDDs. New agents in development will broaden the utility of this approach to other NDDs.

**Table 3:** Summary of complement drugs used in NDDs including preclinical and clinical studies

Disease	Drug name	Type	Target	Stage	Company
**NMO**	Eculizumab	mAb	C5 cleavage	In clinic 2019	Alexion/AZ
	Ravulizumab	mAb	C5 cleavage	Approved in EU; awaiting final FDA approval	Alexion/AZ
	Zilucoplan	Cyclic peptide	C5 cleavage	Pre-trial	UCB
**ALS**	Pegcetacoplan	Linear peptide	C3 cleavage	Phase II trial discontinued (NCT04579666)	Annexon
	ANX-005	mAb	C1q blocker	Phase II trial (NCT04569435)	Apellis
	Ravulizumab	mAb	C5 cleavage	Phase III failed 2021 (NCT04248465)	Alexion/AZ
	Zilucoplan	Cyclic peptide	C5 cleavage	Phase II/III discontinued (NCT04436497)	UCB
	CP010 (anti-C6)	mAb	C6 blocker	Preclinical	Complement Pharma
**HD**	ANX-005	mAb	C1q blocker	Phase II trial (NCT04514367)	Annexon
**MS**	None; No tested anti-complement drugs	Many tested in models	n/a	All preclinical	n/a
**AD**	None; No tested anti-complement drugs	Some data from models	n/a	All preclinical	n/a
**PD**	None; No tested anti-complement drugs	Minimal model data	n/a	All preclinical	n/a
**Schz**	None; No tested anti-complement drugs	Minimal model data	n/a	All preclinical	n/a
**Tauopathies**	None; No tested anti-complement drugs	Minimal model data	n/a	All preclinical	n/a

Abbreviations: AD, Alzheimer’s disease; ALS, Amyotrophic lateral sclerosis; HD, Huntington’s disease; mAb, monoclonal antibodies; MS, multiple sclerosis; NMO, neuromyelitis optica; PD, Parkinson’s disease; Schz, Schizophrenia.

### Complement therapeutics: current anti-complement drugs for brain diseases

Several of the current crop of complement inhibitors, developed to treat complement dysregulation in peripheral diseases, are being evaluated for treatment of NDDs (Table 3). The anti-C5 (mAb) eculizumab, already in the clinic for more than 16 years for the treatment of rare systemic complement dysregulation diseases, was FDA approved for the treatment of the neuromuscular conduction disorder myasthenia gravis (MG) in 2017 and for the demyelinating disease NMO spectrum disorder (NMOSD) in 2019 [219,220]. These successes raise the prospect of using complement inhibitors to treat NDDs currently lacking effective treatments.

Amyotrophic lateral sclerosis (ALS) is a progressive NDD characterized by loss of motor neurones in the brain and spinal cord. Blood–spinal cord barrier (BSCB) and BBB degradation occurs early in ALS, provoking the testing of non-brain penetrant anti-complement drugs, including the C3-blocking linear peptide pegcetacoplan (Apellis) [221], C5-blocking peptide zilucoplan and the anti-C5 mAb ravulizumab [222,223]. Disappointingly, none of these continued beyond Phase II and trials were discontinued [221]. The anti-C1q mAb ANX-005 (Annexon) is currently in Phase IIb for ALS, was well tolerated and the trial should report in 2024 [147].

NMOSD is a demyelinating disease specifically targeting optic tracts and spinal cord; here too the BBB is disrupted early in the disease process permitting access of drugs from the periphery. The C5-blocking mAb eculizumab was FDA approved for NMOSD in 2019 and has become the standard for therapy [162,221,224]. The next-generation, extended half-life version of eculizumab, ravulizumab is also now in the clinic for NMOSD.

### Complement therapeutics in AD

To date, only a handful of drugs are approved for treatment of AD; most of these provide only symptomatic relief and have no impact on disease course [225–229]. In the quest for disease-modifying drugs, most attention has focussed on removing amyloid, culminating in the recent FDA approval of the anti-Aβ antibodies aducanumab and lecanemab despite limited efficacy [230–233]. Lecanemab effectively eliminated amyloid (68% clearance when compared with placebo) but its impact on cognitive decline was modest and side effects were common [234]. The modest effect on cognition despite efficient amyloid clearance reveals a disconnect between amyloid and disease, suggesting the need for alternative treatment strategies. Inhibiting neuroinflammation provides an alternative approach to therapy of AD and complement is an obvious target. Complement inhibition has proved effective in several mouse AD models: inhibition of MAC assembly reduced synapse loss [31,78,108,235]; blocking C5aR1 with the antagonist PMX53 prevented C5a-mediated neuronal death [236,237] and reduced amyloid, microglial activation and inflammation in Tg2576 and 3xTg AD mice [84,117]; C5aR1-antagonist EP67 reduced amyloid load and synapse loss in 5xFAD mice [238].

Despite the above evidence, it has been difficult to convince Pharma that anti-complement drugs should be trialled in AD. Anti-complement drugs have been in the clinic for systemic diseases for more than 15 years; the anti-C5 monoclonal antibody (mAb) Eculizumab is approved for therapy of two neurological conditions, MG and NMOSD [224]. Modifying and repurposing current anti-complement drugs for therapy of AD should be prioritized.

### Complement therapeutics in MS

Current MS treatments use mAbs to target B cells expressing the cell-specific marker CD20; these are effective in most MS subtypes, with the exception of ppMS which responds poorly or not at all to these biological therapies [175]. Despite the evidence above implicating complement in MS and the demonstration that systemic complement blockade is effective in animal models [219,239], no attempts have been made to use currently available complement-blocking drugs for treatment of MS [221]. This is particularly surprising given that anti-complement therapy has proven highly effective in NMOSD, a disease that, although localized to optic nerve and spinal cord, resembles MS in many ways. Eculizumab, the C5 blocking mAb was FDA-approved for NMOSD in 2019 and has become the gold standard [162,221,224]. Trials in MS sub-groups selected based on failure to respond to existing drugs and evidence of complement dysregulation, are overdue.

### Complement in therapeutics schizophrenia

Schizophrenia symptoms are managed with antipsychotics but responses are variable and often poor. Whether targeting complement and inflammation can be beneficial remains to be tested. Evidence from mouse models is sparse, in part due to the lack of good models, and precisely how complement contributes to the disease is unclear. Nevertheless, the above evidence implicating complement as a pathological factor raises the possibility that complement-blocking drugs may ameliorate the course of the illness in some patients.

### Complement therapeutics for NDDs: optimizing the drugs and selecting the patients

In most NDDs the BBB is intact, at least in early disease, preventing access of drugs from the periphery; for example, access for systemically delivered mAb or other large drugs is minimal (<0.1% of peripheral levels). Efficacy of systemically delivered drugs in ALS and NMOSD are the exceptions to this rule because the BBB is impaired early. In ALS patients, BBB dysfunction is evidenced by infiltration of antibodies and other large proteins (e.g., thrombin, plasminogen and hemoglobin), leading to pericyte degeneration and tight junction dysfunction. Albumin (∼67 kDa), a protein typically excluded by the intact BBB, has been observed in the spinal cord and brain tissues of MND patients and is used as a marker of BBB leakage [240–242]. CSF biomarkers for MND include immunoglobulins and albumin, as well as specific markers like GFAP, NSE, and S100 beta proteins, demonstrating an impairment of BBB integrity [243]. For other NDDs, the BBB represents a considerable hurdle for drugs and none of the complement blocking drugs in use today cross the BBB. Current efforts to design and develop BBB-penetrant anti-complement drugs for the early treatment of diverse NDDs are extensively reviewed elsewhere [221].

The second issue to be resolved is patient selection. The different NDDs are all heterogenous conditions and selecting those diseases and cases where complement dysregulation is a driver is key to success in clinical trials and subsequent therapeutic use. Patient stratification and selection is thus a core consideration when designing a clinical study, choosing those with evidence of complement dysregulation for inclusion. For instance, in the trial of the anti-C1q mAb ANX-005 in HD, retrospective analysis showed that a subset of patients with high C4/C4a ratios (indicative of classical pathway activation) responded best to the drug (75% vs. 36% response) [244]. It is thus essential that patients are selected and monitored using tests to measure markers of complement dysregulation in blood and brain, something that has been lacking in trials to date.

In the recent clinical trials (NCT04569435), ANX-005 reduced neurotoxicity and preserve synapses, was well-tolerated in both healthy individuals and the patient populations, with some patients undergoing treatment for up to a year, and robust target engagement was demonstrated in blood, brain and eye [245,246]. To date a handful of other anti-complement drugs, including C3 blockers and anti-C5 agents, have been tested in ALS with disappointing outcomes. Carefully designed clinical trials are required to identify responder subgroups with pronounced complement dysregulation. Measurement of plasma and CSF complement activation products (e.g., TCC, Ba, C3 or C4 fragments, C5a) show promise and may improve the efficiency of future clinical trials. The ongoing clinical trials of anti-complement drugs in NDDs, most in the early phases, are summarized in Table 3. Preliminary results from some trials have shown encouraging outcomes, supporting the potential therapeutic value of complement-targeted drugs. A major challenge that needs to be addressed for the effective targeting of complement in NDDs is the lack of understanding of underpinning mechanisms: When and how is complement dysregulation initiated? Which pathways are involved? Where is complement coming from? What are the main targets of complement attack? Addressing these questions will enable proper targeting of the relevant pathways at the right time in the disease process as new brain-penetrant complement-blocking drugs emerge for therapy of NDDs.

## Data Availability

This is a review article with no primary data so not applicable.

## Open Access

Open access for this article was enabled by the participation of Cardiff University in an all-inclusive *Read & Publish* agreement with Portland Press and the Biochemical Society under a transformative agreement with JISC.

## Abbreviations

αSyn: Alpha synuclein
AD: Alzheimer’s disease
AGD: argyrophilic grain disease
ALS: amyotrophic lateral sclerosis
APOE: apolipoprotein E
APP: amyloid precursor protein
BBB: blood–brain barrier
BSCB: blood–spinal cord barrier
C1-INH: C1 inhibitor
C1qBP: C1q-binding protein
C9orf72: chromosome 9 open reading frame 72
CAA: cerebral amyloid angiopathy
CBD: corticobasal degeneration
CR1: complement receptor 1
CR3: complement receptor 3
CSF: cerebrospinal fluid
CSMD1: CUB and sushi multiple domains 1
DA: dopaminergic
DAF: decay accelerating factor
DLB: dementia with Lewy bodies
DS: Down’s syndrome
FB: factor B
Fcn: ficolin
FH: factor H
FTD: frontotemporal dementia
FUS: fused in sarcoma
GGT: globular glial tauopathy
GRN: progranulin
GWAS: Genome Wide Association Study
HD: Huntington’s disease
ICH: intracerebral haemorrhage
IHC: immunohistochemistry
IPAD: intramural periarterial drainage
iPSC: induced pluripotent stem cell
KO: knockout
LOAD: late onset AD
LPS: lipopolysaccharide
mAb: monoclonal antibody
MAC: membrane attack complex
MAPT: microtubule-associated protein tau
MASP: Mannan-binding lectin-associated serine protease
MBL: Mannan-binding lectin
MCI: mild cognitive impairment
MCP: membrane cofactor protein
MG: Myasthenia Gravis
MHC: major histocompatibility complex
MOG: myelin oligodendrocyte glycoprotein
MOGAD: MOG-associated disorder
MS: multiple sclerosis
MSA: multiple system atrophy
NDD: neurodegenerative disease
NFL: neurofilament light chain
NMOSD: neuromyelitis optica spectrum disorder
NMO: neuromyelitis optica
PBMC: peripgeral blood mononuclear cell
PD: Parkinson’s disease
PiD: Pick’s disease
ppMS: primary progressive MS
PSP: progressive supranuclear palsy
SVD: small vessel disease
TCC: terminal complement complex
TDP-43: transactive response DNA-binding protein
VaD: vascular dementia
VTR: vitronectin
WM: white matter
