# Correction: The complement system in neurodegenerative diseases

**DOI:** 10.1042/CS-2023-0513C_COR

**Published:** 2024-08-14

**Keywords:** complement, dementia, inflammation

The authors of the review article “The complement system in neurodegenerative diseases” (DOI: 10.1042/CS20230513) would like to correct their paper. The authors state that in Table 3 rows 5 and 6, in the “Company” column the words “Annexon” and “Appellis” were inadvertently switched. Additionally, the authors would like to correct an error in the sixth sentence of the “Complement therapeutics in AD” paragraph of their paper. The section “C5aR1-antagonist EP67 reduced amyloid load and synapse loss in 5xFAD mice” should read: “C5aR1-agonist EP67 reduced amyloid load and synapse loss in 5xFAD mice”.

**Table 3:** Summary of complement drugs used in NDDs including preclinical and clinical studies

Disease	Drug Name	Type	Target	Stage	Company
**NMO**	Eculizumab	mAb	C5 cleavage	In clinic 2019	Alexion/AZ
	Ravulizumab	mAb	C5 cleavage	Approved in EU; awaiting final FDA approval	Alexion/AZ
	Zilucoplan	Cyclic peptide	C5 cleavage	Pre-trial	UCB
**ALS**	Pegcetacoplan	Linear peptide	C3 cleavage	Phase II trial discontinued NCT04579666	Apellis
	ANX-005	mAb	C1q blocker	Phase II trial NCT04569435	Annexon
	Ravulizumab	mAb	C5 cleavage	Phase III failed 2021 NCT04248465	Alexion/AZ
	Zilucoplan	Cyclic peptide	C5 cleavage	Phase II/III discontinued NCT04436497	UCB
	CP010 (anti-C6)	mAb	C6 blocker	Preclinical	Complement Pharma
**HD**	ANX-005	mAb	C1q blocker	Phase II trial NCT04514367	Annexon
**MS**	None; No tested anti-complement drugs	Many tested in models	n/a	All preclinical	n/a
**AD**	None; No tested anti-complement drugs	Some data from models	n/a	All preclinical	n/a
**PD**	None; No tested anti-complement drugs	Minimal model data	n/a	All preclinical	n/a
**SZ**	None; No tested anti-complement drugs	Minimal model data	n/a	All preclinical	n/a
**Tauopathies**	None; No tested anti-complement drugs	Minimal model data	n/a	All preclinical	n/a

Abbreviations: mAb, monoclonal antibodies; NMO, Neuromyelitis optica; ALS, Amyotrophic lateral sclerosis; HD, Huntington's disease; MS, multiple sclerosis; HD, Huntington's disease; AD, Alzheimer's disease; PD, Parkinson's disease; Sz, Schizophrenia

The requested correction has been assessed and agreed by the Editorial Board. The authors apologise for this error and declare that this correction does not change the conclusions of the paper. The corrected version of Table 3 is presented here.

